# Mixed viral infections uncover multifaceted RNA silencing strategies in plant antiviral defense

**DOI:** 10.1186/s12864-026-13141-8

**Published:** 2026-07-07

**Authors:** Patrick Materatski, Joana Amaro Ribeiro, Cristiana Marques, André Albuquerque, Maria do Rosário Félix, Carla Varanda

**Affiliations:** 1 Research Centre for Natural Resources, Environment and Society (CERNAS), Santarém Polytechnic University, School of Agriculture, Quinta Do Galinheiro - S. Pedro, Santarém, 2001-904 Portugal; 2 MED – Mediterranean Institute for Agriculture, Environment and Development, CHANGE – Global Change and Sustainability Institute,, Universidade de Évora, Largo Dos Colegiais 2, Évora, 7004-516 Portugal; 3 MED – Mediterranean Institute for Agriculture, Environment and Development, CHANGE – Global Change and Sustainability Institute, Departamento de Fitotecnica, Escola de Ciências e Tecnologia, Universidade de Évora, Largo Dos Colegiais 2, 7004-516 Évora, Portugal

**Keywords:** Viral single and mixed-infections, *Alphanecroviruses*, DCLs, Gene silencing, VsiRNAs

## Abstract

**Background:**

*Alphanecrovirus tessellati* and *Alphanecrovirus oleae* are closely related alphanecroviruses, sharing over 74% genomic identity and frequently co-infecting plants under natural conditions. Despite their frequent coexistence, the molecular consequences of single versus mixed infections, particularly concerning host RNA silencing responses, remain poorly understood. Here, we investigated the antiviral defense responses of *Nicotiana benthamiana* to single and mixed infections with both viruses, focusing on symptom development, viral accumulation, *Dicer-like* (*DCL*) gene expression, and virus-derived small interfering RNA (vsiRNA) profiles.

**Results:**

Single infections resulted in contrasting disease phenotypes: *A. oleae* induced severe systemic necrosis and high viral accumulation, whereas *A. tessellati* caused transient infections with mild symptoms. In mixed infections, *A. tessellati* accumulated to significantly higher levels and was associated with enhanced disease severity, indicating that the interaction between the two viruses altered infection dynamics and favored *A. tessellati* accumulation. In contrast, *A. oleae* showed reduced relative genomic RNA accumulation in mixed infections despite the persistence of disease symptoms. Gene expression analyses showed consistent upregulation of *DCL4* and *DCL2* during mixed infection, supporting their involvement in antiviral RNA silencing under co-infection conditions. Small RNA profiling revealed infection-dependent differences in vsiRNA accumulation patterns. Notably, mixed infection was associated with a change in the predominant vsiRNA size class for *A. oleae,* alongside a shift in vsiRNAs genomic targeting from the RdRp gene during single infection to the suppressor-associated p6 gene during mixed infection, indicating a reprogramming of host silencing responses.

**Conclusions:**

Our results demonstrate that closely related viruses can differentially modulate host RNA silencing responses depending on the infection context. These findings highlight the plasticity of antiviral RNA silencing during viral co-infections and provide new insights into how mixed infections reshape host-virus interactions at the molecular level.

**Supplementary Information:**

The online version contains supplementary material available at https://doi.org/10.1186/s12864-026-13141-8.

## Background

*Alphanecrovirus oleae* and *Alphanecrovirus tessellati*, formerly called *Olive latent virus 1* (OLV-1) and *Olive mild mosaic virus* (OMMV), respectively, belong to the genus *Alphanecrovirus*, family *Tombusviridae*. *A. tessellati* and *A. oleae* present a positive-sense single-stranded RNA (∼3.7 kb) encoding five proteins: *p23* (a *pre-readthrough protein*); *p82* (the *RNA-dependent RNA polymerase*, *RdRp*); *p8* and *p6* (*small proteins*); and the coat protein (CP); *p29* in *A. tessellati* and *p30* in *A. oleae* [1, 2]. Most of the protein functions have been characterized for both viruses: *p23* and *RdRp* are involved in viral RNA replication; *p8* and *p6* are predicted to participate in virus movement, and *CP* is involved in capsid assembly, systemic movement, and vector transmission [3–6]. In *A. tessellati*, *p6* and *CP* have been reported to function as silencing suppressors [2]. No viral suppressors have been described for *A. oleae*. The highest sequence similarity between *A. tessellati* and *A. oleae* lies in the *p6*, *p8*, and *RdRp* regions, 100%, 87.6% and 92.1%, respectively [7]. *A. tessellati* and *A. oleae* were first identified in olive trees (*Olea europaea* L.) [4, 8] where they frequently appear in mixed infections [9, 10]. In fact, the origin of *A. tessellati* has been suggested as a result from recombination events involving *A. oleae* and the Tombusvirus *Betanecrovirus nicotianae* (former TNV-D) during co-infection [9, 11].

Viral mixed infections can significantly change viral population dynamics, host immune responses and the course of disease progression in plants and are widespread in both agricultural and wild systems, although in agricultural systems the mixed infections can significantly decrease the crop yields [12, 13]. Depending on virus–virus and virus–host interactions, outcomes may be synergistic (e.g., enhanced symptoms or viral load), antagonistic (mutual suppression), or neutral [14, 15]. Synergy may arise through molecular complementation or one virus benefiting from another's VSRs, whereas antagonism may result from resource competition, spatial exclusion, or activation of divergent silencing pathways [16].

In olive, *A. oleae* and *A. tessellati* typically cause mild or no apparent symptoms; however, mixed infections have been associated with an intensification of symptoms and an increase in transmission efficiency [9, 10, 17, 18]. *A. oleae* is easily acquired by roots of *N. benthamiana*, whereas *A. tessellati* soil transmission is less efficient, and in nature, it must rely on its biological vectors *Olpidium brassicae* and *Olpidium virulentus* [6, 18]. In the reported study, *A. tessellati* was able to invade plant roots, in the presence of *A. oleae* and in the absence of a biological vector, suggesting a facilitative interaction [17, 18].

In addition, recent HTS data indicated that the simultaneous presence of multiple viruses (e.g., *Olive leaf yellowing-associated virus* (OLYaV) and *Olive leaf mottling virus* (OLMV)) may be associated with complex symptoms such as mosaic, chlorosis, and defoliative shedding [19]. These *Alphanecroviruses* have also been detected in non-olive hosts; *A. oleae* has been detected in citrus trees [20] and tulips [21], the latter where *A. tessellati* has also been identified as part of the viral complex associated with the Augusta disease, previously ascribed to TNV [22]. As for economically important horticultural crops, *A. oleae* has been reported in tomato [23, 24] and *A. tessellati* in spinach [25] and corn salad [26]. Both viruses are mechanically transmitted to leaves of *N. benthamiana,* where they induce chlorotic and necrotic lesions, often resulting in plant death [17, 20]. Although natural co-infection of these *Alphanecrovirus* species in horticultural crops has not yet been reported, it is anticipated, given their biological similarities and overlapping host potential.

From the host side, a key component of the plant immune system against RNA viruses is post-transcriptional gene silencing (PTGS), also referred to as RNA silencing [27]. This defense mechanism relies on the recognition and processing of viral double-stranded RNA (dsRNA) intermediates by *Dicer-like* (*DCL*) proteins [28]. The *DCLs* -*RNase III* enzymes process double-stranded viral RNA into small interfering RNAs (siRNAs), referred to as virus-derived small interfering RNAs (vsiRNAs), which are then incorporated into *Argonaute* (*AGO*) proteins, forming RNA-induced silencing complexes (RISCs) that degrade homologous guided by the vsiRNA [28, 29]. The DCL family in plants typically includes *DCL1*–*DCL4*, each with distinct but sometimes overlapping roles. *DCL4* is considered the most potent class for antiviral defense and primarily generates 21-nt siRNAs, while *DCL2* produces 22-nt siRNAs, especially when *DCL4* is absent or overwhelmed [30, 31]. *DCL3* is mainly associated with 24-nt siRNAs involved in transcriptional gene silencing via DNA methylation, but it can be engaged during viral infections as well [32]. *DCL1* primarily processes microRNAs (miRNAs) and has a minor role in antiviral defense.

To overcome plant defense mechanisms and ensure successful replication and disease development, viruses have evolved several strategies. Among these, viral suppressors of RNA silencing (VSRs) represent one of the most efficient mechanisms and involve virus-encoded proteins that inhibit the plant RNA silencing pathway [33, 34]. The suppressor proteins interfere in different stages of the RNA silencing pathways, being highly divergent and appearing to have evolved independently in the different viruses. These suppressors bind dsRNAs inhibiting their processing into siRNAs; in addition, they are capable of sequestering vsiRNAs, further inhibiting their incorporation into RISC or directly interfering with the recognition of viral RNA, dicing, and RISC assembly [35]. In mixed infections, interactions between different VSRs and their combined effects on the host silencing system can result in distinct disease outcomes, ranging from enhanced resistance to systemic viral synergy, depending on the specific host-virus combination and environmental conditions [36]. Studies in *N. benthamiana* show that most RNA viruses produce abundant 21-nt or mixed 21/22-nt vsiRNAs [37, 38]. Exceptions include *Tomato spotted wilt virus* (TSWV) and *Rice stripe virus* (RSV), which show notable 24-nt dominance [39, 40]. A few reports describe increased 22-nt vsiRNAs or overall abundance during mixed infections involving satellite RNAs [41–44], however, to date, no study has reported a qualitative shift in vsiRNA genomic targeting between single and mixed infections for RNA viruses, namely a change in the predominant targeted viral genes or the emergence of new vsiRNA hotspots within the same gene.

Many synergistic interactions have been recognized to contribute to increased pathogenicity and breakdown of host resistance [14, 45–48]. Yet, the molecular underpinnings of this synergism, especially regarding silencing suppression and vsiRNA biogenesis, remain unexplored. In this study, we hypothesize that *N. benthamiana* mounts distinct antiviral responses against *A. tessellati* and *A. oleae* during single and mixed infections. We evaluated viral accumulation and symptom progression across time points, examined DCL gene expression, and profiled the biogenesis of vsiRNAs via small RNA deep sequencing. Our goal was to determine whether viral mixed-infection reshapes host RNA silencing responses, alters vsiRNA profiles, and redirects targeting patterns across viral genomes.

## Results

### Mixed-infection increases symptom severity in *N. benthamiana*

**Fig. 1:**
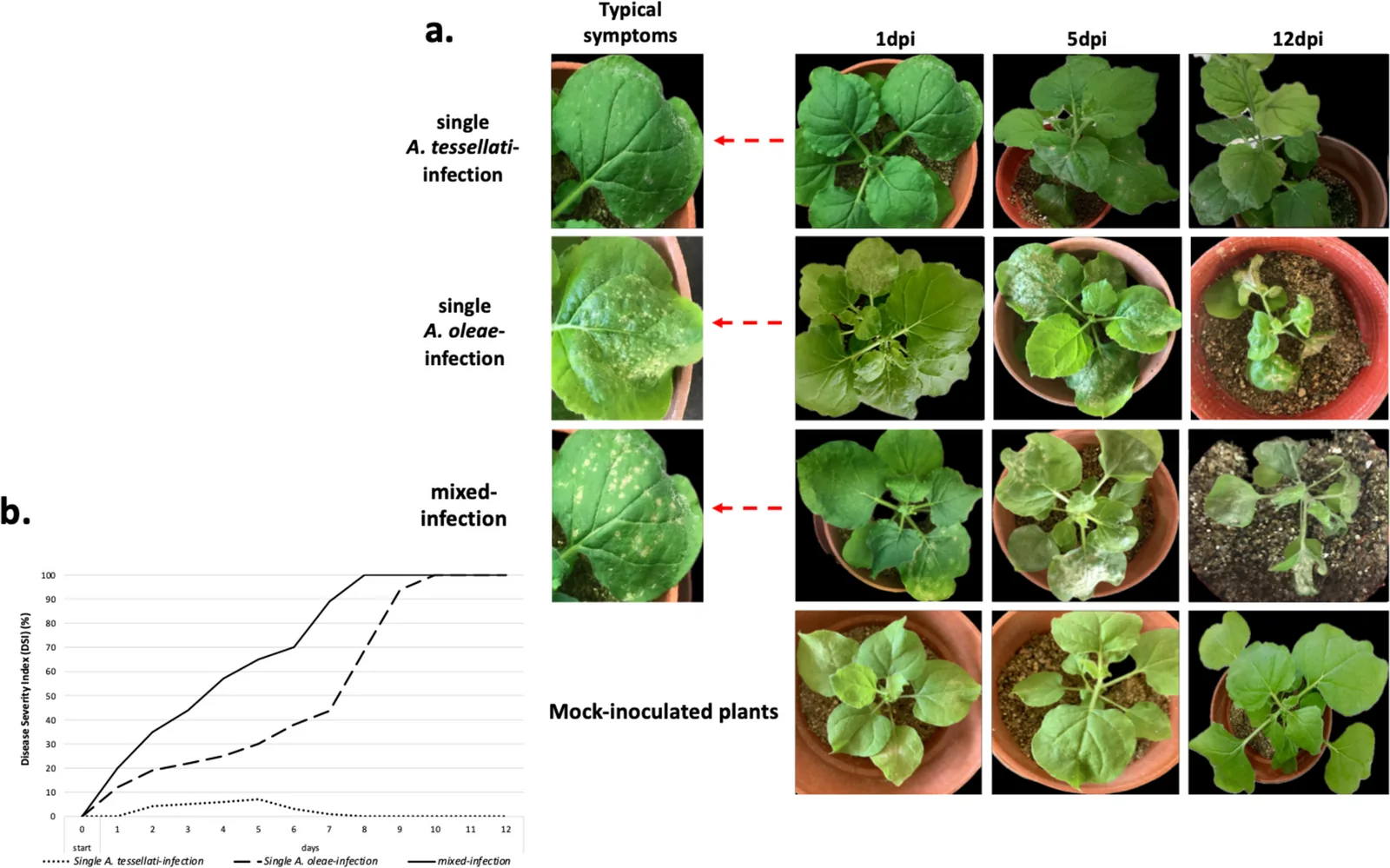
Visual observation of leaves from *N. benthamiana* plants. **a** single infected with *A. tessellati*, *A. oleae* and *A. tessellati* and *A. oleae* mixed-infected at 1 dpi, 5 dpi, and 12 dpi. Control plants were inoculated with sodium phosphate buffer without the viruses presence. **b** Disease Severity Index at 0 (beginning of the assay), 12 h and 1 to 12 days after single and mixed-infection of *A. tessellati* and *A. oleae* in *N. benthamiana* plants

Disease progression in *N. benthamiana* was assessed using a 0–4 Disease Severity Index (DSI) scale (Fig. 1a, b). Mock-inoculated plants remained asymptomatic throughout the experiment. All virus-inoculated plants developed visible symptoms between 1 and 2 dpi, with clear differences in symptom progression depending on the infection type. Single infection with *A. tessellati* resulted in mild and transient symptoms, characterized by local chlorotic mosaic lesions appearing at 2 dpi (DSI: 4%). Disease severity peaked at 5 dpi (7%) and subsequently regressed, with full recovery observed by 12 dpi. In contrast, *A. oleae* single infection caused rapid disease progression, with symptoms appearing at 1 dpi and evolving from local chlorotic lesions to severe necrosis. Systemic symptoms became evident by 5dpi, culminating in complete disease severity by 12 dpi and substantial plant mortality thereafter. Mixed infection accelerated symptom development and increased disease severity. Chlorotic lesions appeared at 1 dpi with higher initial DSI values than in single infections, followed by a rapid progression to maximal disease severity by 8 dpi. Mixed infections were associated with higher mortality rates compared to single infections. These results demonstrate that mixed infection both accelerates disease development and exacerbates symptom severity, leading to earlier plant death.

### Enhanced accumulation of *A*. *tessellati* and reduced relative abundance of *A*. *oleae *in mixed infections

**Fig. 2:**
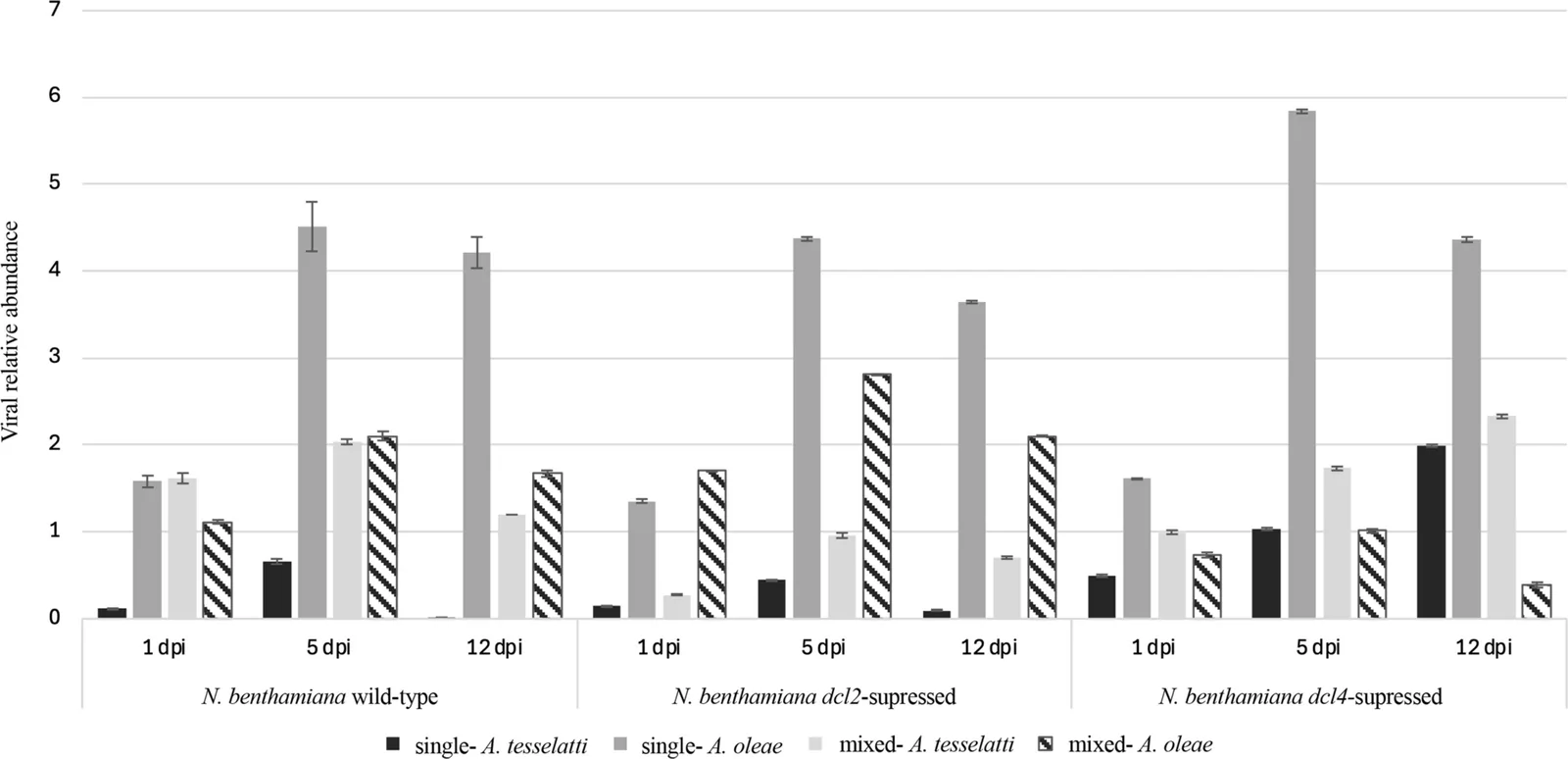
Mean relative abundance ± standard error (SE) of single *A. tessellati*-infection, single *A. oleae*-infection *A. tessellati* and *A. oleae* mixed-infections at each time points (1dpi, 5dpi and 12 dpi) in Wild-type *N. benthamiana* plants and *dcl2* and *dcl4*-suppressed plants. Additionally, detailed results from complementary PERMANOVA statistical analyses are presented in Table 1

Relative viral genomic RNA accumulation was quantified by qRT-PCR. In wild type plants, both viruses reached peak accumulation at 5 dpi in single and mixed infections. In single infections, *A. tessellati* showed transient accumulation peaking at 5 dpi, whereas *A. oleae* maintained high accumulation at both 5 and 12 dpi. In mixed infections, *A. tessellati* accumulated at significantly higher levels than in single infection at all evaluated time points, although its accumulation within mixed infection peaked at 5 dpi and subsequently decreased at 12 dpi. Conversely, *A. oleae* showed consistently lower relative accumulation in mixed infections compared to single infections, despite sustained disease symptoms. At 5 dpi, both viruses accumulated to comparable levels during mixed infection. Together, these data indicate that mixed infection altered the relative accumulation patterns of both viruses, favoring the accumulation of *A. tessellati*, whereas *A. oleae* showed comparatively lower accumulation levels under mixed-infection conditions. Despite this, the presence of both viruses was associated with accelerated symptom development and increased disease severity in co-infected plants (Fig. 2; Table 1).

**Table 1:** Pairwise PERMANOVA comparisons of viral relative abundance in wild-type *N. benthamiana* plants

a. Temporal comparisons within treatments (p-values)			
	**1 dpi *vs* 5 dpi**	**1 dpi *vs* 12 dpi**	**5 dpi *vs* 12 dpi**
***N. benthamiana* Wild-type**	***p* values**		
single-*A. tessellati*	0.0001	0.0001	0.0001
single-*A. oleae*	0.0002	0.0001	0.1017
mixed-*A. tessellati*	0.0051	0.0013	0.0001
mixed-*A. oleae*	0.0005	0.0001	0.0001

**b. Comparisons among treatments at each time point (p-values)**			
	**1 dpi**	**5 dpi**	**12 dpi**
***N. benthamiana* Wild-type**	***p* values**		
single-*A. tessellati vs* mixed-*A. tessellati*	0.0001	0.0046	0.0001
single-*A. tessellati vs* single-*A. oleae*	0.0001	0.0001	0.0002
single-*A. tessellati vs* mixed-*A. oleae*	0.0001	0.0001	0.0001
mixed-*A. tessellati vs* single-*A. oleae*	0.0003	0.0001	0.0001
mixed-*A. tessellati vs* mixed-*A. oleae*	0.0001	0.0001	0.0001
single-*A. oleae vs* mixed-*A. oleae*	0.0001	0.378	0.0001

**c. Temporal comparisons within treatments (p-values)**			
	**1 dpi *vs* 5 dpi**	**1 dpi *vs* 12 dpi**	**5 dpi *vs* 12 dpi**
***N. benthamiana* DCL2 supressed**	***p* values**		
single-*A. tessellati*	0,0002	0,0341	0,0003
single-*A. oleae*	0,0001	0,0001	0,0001
mixed-*A. tessellati*	0,0001	0,0001	0,0009
mixed-*A. oleae*	0,0001	0,0001	0,0001

**d. Comparisons among treatments at each time point (*p*-values)**			
	**1 dpi**	**5 dpi**	**12 dpi**
***N. benthamiana* DCL2 supressed**	***p* values**		
single-*A. tessellati vs* mixed-*A. tessellati*	0,0023	0,0001	0,0001
single-*A. tessellati vs* single-*A. oleae*	0,0001	0,0001	0,0001
single-*A. tessellati vs* mixed-*A. oleae*	0,0001	0,0001	0,0001
mixed-*A. tessellati vs* single-*A. oleae*	0,0001	0,0002	0,0001
mixed-*A. tessellati vs* mixed-*A. oleae*	0,0001	0,0001	0,0001
single-*A. oleae vs* mixed-*A. oleae*	0,0002	0,0001	0,0001

**e. Temporal comparisons within treatments (*p*-values)**			
	**1 dpi *vs* 5 dpi**	**1 dpi *vs* 12 dpi**	**5 dpi *vs* 12 dpi**
***N. benthamiana* DCL4 supressed**	***p* values**		
single-*A. tessellati*	0,0001	0,0001	0,0001
single-*A. oleae*	0,0001	0,0001	0,0001
mixed-*A. tessellati*	0,0001	0,0001	0,0001
mixed-*A. oleae*	0,0015	0,0021	0,0005

**f. Comparisons among treatments at each time point (*p*-values)**			
	**1 dpi**	**5 dpi**	**12 dpi**
***N. benthamiana* DCL4 supressed**	***p* values**		
single-*A. tessellati vs* mixed-*A. tessellati*	0,0001	0,0001	0,0003
single-*A. tessellati vs* single-*A. oleae*	0,0001	0,0001	0,0001
single-*A. tessellati vs* mixed-*A. oleae*	0,0008	0,4807	0,0001
mixed-*A. tessellati vs* single-*A. oleae*	0,0001	0,0001	0,0001
mixed-*A. tessellati vs* mixed-*A. oleae*	0,0021	0,0001	0,0001
single-*A. oleae vs* mixed-*A. oleae*	0,0001	0,0001	0,0001

**a**. Temporal comparisons (1 vs 5 dpi, 1 vs 12 dpi, and 5 vs 12 dpi) within treatments (single *A. tessellati*-infection; single *A. oleae*-infection; mixed *A. tessellati*-infection; and mixed *A. oleae*-infection); **b**. Comparisons among treatments (single-*A. tessellati* vs mixed-*A. tessellati*; single-*A. tessellati* vs single-*A. oleae*; single-*A. tessellati* vs mixed-*A. oleae*; mixed-*A. tessellati* vs single-*A. oleae*; mixed-*A. tessellati* vs mixed-*A. oleae*; and single-*A. oleae* vs mixed-*A. oleae*) at each time point (1, 5, and 12 dpi). Pairwise PERMANOVA comparisons of relative viral abundance in *dcl2*-suppressed plants: **c** Temporal comparisons (1 vs 5 dpi, 1 vs 12 dpi, and 5 vs 12 dpi) within treatments (single *A. tessellati*-infection; single *A. oleae*-infection; mixed *A. tessellati*-infection; and mixed *A. oleae*-infection); **d** Comparisons among treatments (single-*A. tessellati* vs mixed-*A. tessellati*; single-*A. tessellati* vs single-*A. oleae*; single-*A. tessellati* vs mixed-*A. oleae*; mixed-*A. tessellati* vs single-*A. oleae*; mixed-*A. tessellati* vs mixed-*A. oleae*; and single-*A. oleae* vs mixed-*A. oleae*) at each time point (1, 5, and 12 dpi). Pairwise PERMANOVA comparisons of relative viral abundance in *dcl4*-suppressed plants: **e** Temporal comparisons (1 vs 5 dpi, 1 vs 12 dpi, and 5 vs 12 dpi) within treatments (single *A. tessellati*-infection; single *A. oleae*-infection; mixed *A. tessellati*-infection; and mixed *A. oleae*-infection); **f** Comparisons among treatments (single-*A. tessellati* vs mixed-*A. tessellati*; single-*A. tessellati* vs single-*A. oleae*; single-*A. tessellati* vs mixed-*A. oleae*; mixed-*A. tessellati* vs single-*A. oleae*; mixed-*A. tessellati* vs mixed-*A. oleae*; and single-*A. oleae* vs mixed-*A. oleae*) at each time point (1, 5, and 12 dpi)

In *dcl2*-suppressed plants, *A. tessellati* accumulation remained low across infection types, indicating that suppression of *DCL2* alone is insufficient to support robust systemic infection by this virus. In contrast, *A. oleae* accumulated efficiently in single infections and dominated mixed infections, establishing systemic infection in all cases. In *dcl4*-suppressed plants, *A. tessellati* accumulated at all time points in single infections and became the dominant virus during mixed infection, surpassing *A. oleae* across all sampling points. These results indicate that *DCL4* plays a key role in restricting *A. tessellati* accumulation and that suppression of *DCL4* shifts viral dominance during mixed infection (Fig. 2; Table 1).

### Dynamic regulation of DCL gene expression across infection contexts

**Fig. 3:**
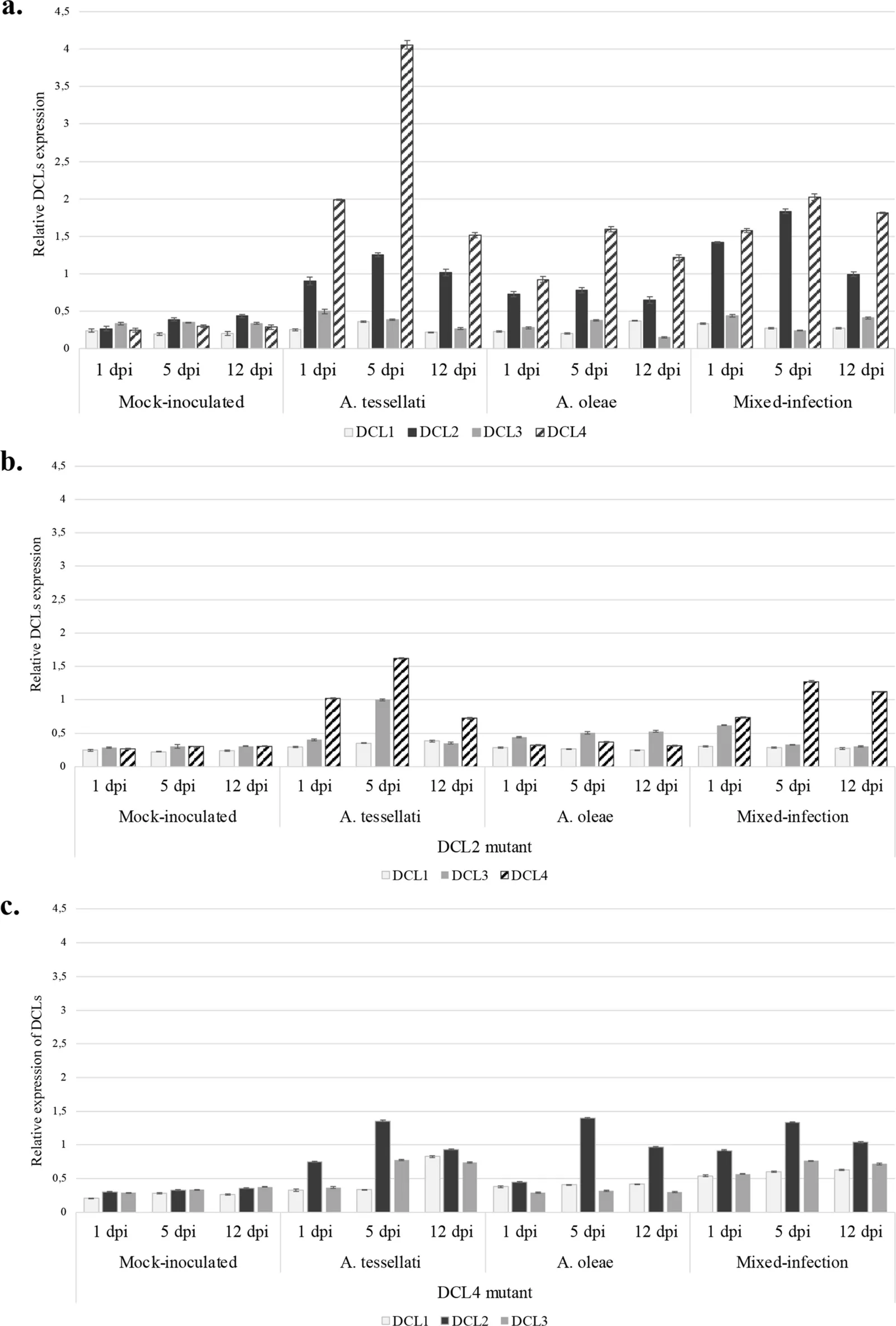
Mean relative expression ± standard error (SE) of DCLs (DCL1–DCL4) genes at each type of infection (mock-inoculated, single *A. tessellati*-infection, single *A. oleae*-infection *A. tessellati* and *A. oleae* mixed-infection) and time points (1dpi, 5dpi and 12 dpi) in Wild-type *N. benthamiana* (**a**.) plants and *dcl2*-suppressed plants (**b**.) and *dcl4*-suppressed plants (**c**.). Additionally, detailed results from complementary PERMANOVA statistical analyses are presented in Table 2 (Wild-type *N. benthamiana*) and Table S1 (*dcl2* and *dcl4*-suppressed plants)

In wild-type plants, *DCL4* expression peaked at 5 dpi across all infection conditions and was consistently higher than *DCL2*. Both genes were upregulated in infected plants relative to mock controls, with mixed infection inducing particularly strong responses at 5 dpi (Fig. 3a; Table 2). *DCL2* expression also peaked at 5 dpi in most infection contexts. Mixed infection promoted significantly higher *DCL2* expression compared to single infections, especially relative to *A. oleae* single infection and early stages of *A. tessellati* infection. Notably, *DCL4* expression in mixed infection was lower than that observed during single *A. tessellati* infection at 5 dpi, suggesting that co-infection may alter the *A. tessellati*-associated *DCL4* response. Overall, these results indicate that mixed infection modulates antiviral RNA silencing responses, characterized by elevated *DCL4* expression and increased *DCL2* responsiveness under co-infection conditions.

**Table 2:** The two-way PERMANOVA test conducted between; “Treatment” (mock-inoculated, single *A. tessellati*-infection, single *A. oleae*-infection *A. tessellati* and *A. oleae* mixed-infection) and “Time points” (1dpi, 5dpi and 12 dpi) using pairwise comparisons performed with 9999 permutations under a reduced model to assess differences between DCLs (DCL1–DCL4) genes in Wild-type *N. benthamiana* plants

Treatments	DCL1			DCL2		
	1dpi	5dpi	12 dpi	1dpi	5dpi	12 dpi
	*p* values			*p* values		
mock-inoculated *vs* single-*A. tessellati*	0.6515	0.0013	0.4969	0.0006	0.0002	0.0001
mock-inoculated *vs* single-*A. oleae*	0.7955	0.5543	0.0104	0.001	0.0002	0.0065
mock-inoculated *vs* mixed infection	0.0302	0.0143	0.0876	0.0004	0.0001	0.0001
single-*A. tessellati vs* single-*A. oleae*	0.2487	0.0001	0.0001	0.0512	0.0012	0.004
single-*A. tessellati vs* mixed infection	0.008	0.0023	0.0016	0.0015	0.0003	0.6696
single-*A. oleae vs* mixed infection	0.0022	0.0032	0.0003	0.0004	0.0001	0.0039
**Treatments**	**DCL3**			**DCL4**		
	1dpi	5dpi	12 dpi	1dpi	5dpi	12 dpi
	*p* values			*p* values		
mock-inoculated *vs* single-*A. tessellati*	0.0109	0.0198	0.0102	0.0001	0.0001	0.0002
mock-inoculated *vs* single-*A. oleae*	0.0798	0.0916	0.0003	0.0001	0.0001	0.0002
mock-inoculated *vs* mixed infection	0.0169	0.0003	0.0137	0.0001	0.0001	0.0002
single-*A. tessellati vs* single-*A. oleae*	0.0016	0.479	0.0013	0.0001	0.0001	0.0056
single-*A. tessellati vs* mixed infection	0.1817	0.0003	0.0017	0.0002	0.0001	0.0017
single-*A. oleae vs* mixed infection	0.002	0.0004	0.0001	0.0002	0.0013	0.0003
**Treatments**	**DCL1**			**DCL2**		
	1 dpi *vs* 5 dpi	1 dpi *vs* 12 dpi	5 dpi *vs* 12 dpi	1 dpi *vs* 5 dpi	1 dpi *vs* 12 dpi	5 dpi *vs* 12 dpi
	*p* values			*p* values		
mock-inoculated	0.1719	0.3298	0.9248	0.0408	0.0158	0.2056
single-*A. tessellati*	0.0032	0.0477	0.0001	0.0064	0.1723	0.0091
single-*A. oleae*	0.0627	0.0008	0.0001	0.3399	0.2323	0.0789
mixed infection	0.0117	0.0084	0.874	0.0004	0.0006	0.0002
**Treatments**	**DCL3**			**DCL4**		
	1 dpi *vs* 5 dpi	1 dpi *vs* 12 dpi	5 dpi *vs* 12 dpi	1 dpi *vs* 5 dpi	1 dpi *vs* 12 dpi	5 dpi *vs* 12 dpi
	*p* values			*p* values		
mock-inoculated	0.4692	0.8447	0.3667	0.1351	0.3495	0.7103
single-*A. tessellati*	0.0232	0.0008	0.0009	0.0001	0.0003	0.0001
single-*A. oleae*	0.0037	0.0005	0.0003	0.0004	0.0071	0.0029
mixed infection	0.0004	0.2692	0.0004	0.0008	0.0011	0.0073

In *dcl2*-suppressed plants, *DCL4* remained the most highly expressed *DCL* during viral infection, particularly in single *A. tessellati* and mixed infections, whereas *DCL3* showed lower expression levels and a more limited response. In contrast, in *dcl4*-suppressed plants, *DCL2* exhibited the highest expression levels across infection types, while *DCL3* displayed moderate expression. However, the expression levels of *DCL4* in the *dcl2* mutant background and *DCL2* in the *dcl4* mutant background were generally lower than those observed in wild-type plants. Overall, these results suggest distinct patterns of *DCL* expression depending on the genetic background, with *DCL2* and *DCL4* remaining the predominant *DCL* transcripts under their respective silencing conditions (Fig. 3b, c; Table S1).

### Mixed infection alters vsiRNAs accumulation and size profiles

Genome comparison performed by Job Dispatcher analysis showed 74.7% similarity between *A. tessellati* and *A. oleae*. Small RNA analysis was conducted at 5 dpi, corresponding to peak symptom severity, viral accumulation, and *DCL* expression. The total number of trimmed sequence reads recovered was 24,961,203 for single *A. tessellati* infection, 11,593,019 for single *A. oleae* infection, and 13,617,985 for mixed infection. Following the assembly of non-host (viral) reads, the total number of vsiRNAs was 5,830,169 for the single *A. tessellati* infection, compared to 2,519,107 for the mixed infection, representing an approximate 50% decrease in vsiRNA abundance. Similarly, vsiRNA counts for *A. oleae* dropped from 1,291,579 in the single infection to 76,791 in the mixed infection, reflecting a nearly 17-fold decrease (Table 3).

**Table 3:** Summary of RNA sequencing performed using the Illumina NovaSeq platform, presenting the total number of reads (18–25 nt) for each infection type (single *A. tessellati* infection, single *A. oleae* infection, and mixed infection with *A. tessellati* and *A. oleae*)

Type of infection		Raw reads	Virus reads	Host reads	vsiRNAs orientation (%)	
					**Forward**	**Reverse**
Single	*A. tessellati*	24 961 203	5 830 169	19 131 034	99.7	0.3
	*A. oleae*	11 593 019	1 291 579	10 301 440	99.7	0.3
Mixed	*A. tessellati*	11 099 051	2 519 107	8 580 116	99.7	0.3
	*A. oleae*	13 541 195	76 791	13 464 404	96.3	3.7

The table includes raw read counts, as well as the number of reads mapped to viral and host genomes. Additionally, the percentage distribution of vsiRNA orientations (forward and reverse) is provided for each infection type

The siRNA orientation profile showed a strong predominance of forward-strand reads (> 99%) in most conditions, indicating clear strand bias. However, *A. oleae* vsiRNAs in mixed infection showed an increased proportion of reverse-strand reads, indicating altered processing dynamics (Table 3). In addition, the number of vsiRNA contact points along the viral genomes decreased during mixed infection, particularly for *A. oleae*, suggesting reduced targeting breadth.

**Fig. 4:**
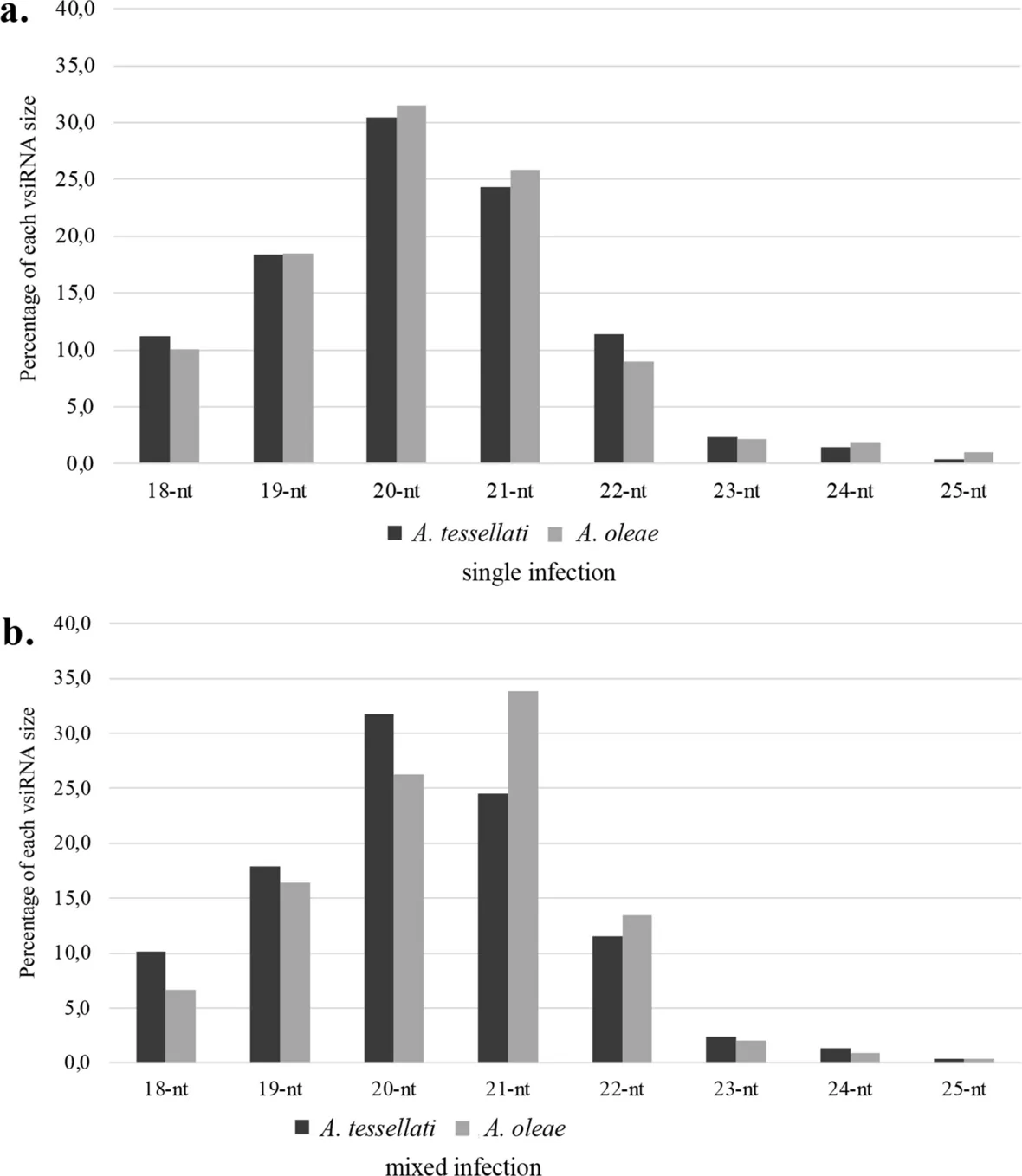
Percentage distribution of vsiRNA size classes (18–25-nt) under different infection conditions: (**a**.) in the single infection of *A. tessellati* and of *A. oleae;* (**b**.) in the mixed-infection of *A. tessellati* and of *A. oleae*

In single infections, vsiRNAs from both viruses were dominated by 20-nt class (30.5% to *A. tessellati* and 31.5% to *A. oleae*), followed by 21-nt (24.3% to *A. tessellati* and 25.8% to *A. oleae*) and 19-nt (18.4% to *A. tessellati* and 18.5% to *A. oleae*) classes (Fig. 4a). A similar pattern was observed for *A. tessellati* during mixed infection to 20-nt, 21nt and 19-nt classes (31.8%, 24.5% and 17.9%, respectively). In contrast, *A. oleae* showed a shift in vsiRNA size distribution under mixed infection, with 21-nt vsiRNAs becoming the most abundant class (33.9%), surpassing 20-nt reads (26.3%) (Fig. 4b).

### Mixed infection redirects vsiRNA genomic targeting

**Fig. 5:**
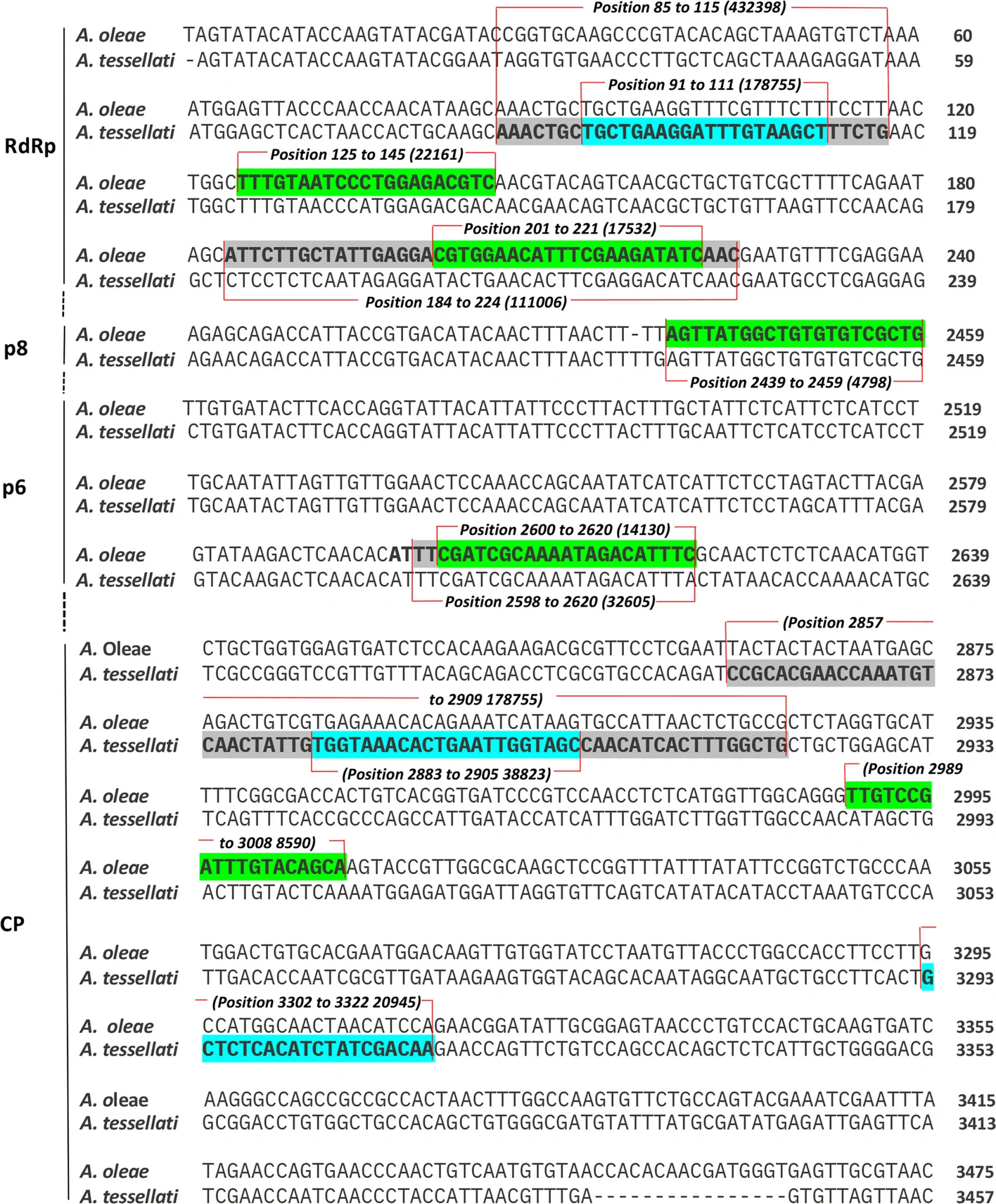
The nucleotides sequences alignment in the higher clived portions of the RdRp, p8, p6 and CP genes of *A. oleae* (DQ083996.1) and *A. tessellati* (HQ651834.1) retrieved from the GenBank database. Blue shaded nucleotides are highlighting the high clived vsiRNAs in the *A. tessellati* genome positions, inluding the number of the reads. Green shaded nucleotides are highlighting the high clived vsiRNAs in the *A. oleae* genome positions, inluding the number of the reads. Gray shaded nucleotides are highlighting all expanded positions in terms of cliveting

In *A. tessellati*, vsiRNA targeting during single infection was predominantly concentrated in the *RdRp*/*p23* region, which represented the major hotspot with 178,755 reads. This hotspot remained conserved during mixed infection, although the overall abundance was reduced to 60,033 reads. Additional targeting was observed in the *CP* gene under both infection conditions (Fig. 5). In *A. oleae*, single infection showed two dominant vsiRNA targeting within the *RdRp*/*p23* region with 22,161 reads and 17,532 reads. In contrast, mixed infection caused a pronounced shift in vsiRNA targeting toward the suppressor-associated p6 gene with 14,130 reads, which became the primary hotspot. Secondary targeting was also detected in the p8 gene with 4,798 reads. These results demonstrate a qualitative reprogramming of vsiRNA targeting in *A. oleae* during mixed infection (Fig. 5; Tables 4, S2).

**Table 4:** Summary of the total number of reads and their respective percentages in the coding genes (p23: readthrough*;* RdRp*:* RNA-dependent RNA polymerase; coat protein; p8: movement protein; p6: movement protein and silence suppressor) at each type of infection (single *A. tessellati*-infection, single *A. oleae*-infection, *A. tessellati* and *A. oleae* mixed-infection)

**Type of**	Gene	**p23**	**RdRp**	**p8**	**p6**	**CP**	**Total reads**	**Total reads**
**infection**	Position in genome	60–668	60–2234	2218–2439	2443–2613	2636–3445	within genes	
*A. tessellati*	Nº of reads	1 567 790	3 606 927	380 539	309 656	1 046 308	5 319 304	5 830 169
	%	**26.9**	**61.9**	**6.5**	**5.3**	**17.9**	**91.2**	100
*A. tessellati*-mixed	Nº of reads	582 294	1 554 372	185 913	142 135	433 385	2 305 621	2 518 935
	%	**23.1**	**61.7**	**7.4**	**5.6**	**17.2**	**91.5**	100
	Gene	**p23**	**RdRp**	**p8**	**p6**	**CP**	genes reads	total reads
	Gene position	61–669	61–2235	2219–2440	2443–2613	2635–3447		
*A. oleae*	Nº of reads	358 344	760 820	75 330	109 385	250 202	1 191 057	1 291 579
	%	**27.7**	**58.9**	**5.8**	**8.5**	**19.4**	**92.2**	100
*A. oleae*-mixed	Nº of reads	10 930	21 301	8754	34 724	5074	69 742	76 791
	%	**14.2**	**27.7**	**11.4**	**45.2**	**6.6**	**90.8**	100

Analysis of the 5′-terminal nucleotide composition revealed that *A. tessellati*-derived vsiRNAs showed similar nucleotide preferences in single and mixed infections, with a predominance of C (32–34.3%), followed by U (30.4–31.4%) and A (25.2–25.5%). Since vsiRNAs carrying a 5′ C, U, and A are preferentially loaded into *AGO5*, *AGO1*, and *AGO2*/*AGO4* complexes, respectively, these results suggest that *AGO* recruitment remained largely stable during mixed infection. In contrast, *A. oleae*-derived vsiRNAs displayed altered nucleotide composition under mixed infection, characterized by an increase in 5′ C (from 30.3% to 50.4%) and a decrease in 5′ A (from 22.7% to 8.7%), suggesting potential shifts in *AGO* loading preferences and changes in host antiviral RNA silencing responses (Table 5).

**Table 5:** Percentages of nucleotide 5’-terminal composition of the vsiRNAs and their associated Argonauts. C nucleotides at 5’-terminal preferentially load into the AGO5 complexes; U nucleotides into the AGO1 complexes and A nucleotides into the AGO2 and AGO4 complexes

Argonauts	Single-infection		Mixed-infection	
	*A. tessellati*	*A. oleae*	*A. tessellati*	*A. oleae*
	Percentage (%)		Percentage (%)	
**AGO-1**	31.4	38.2	30.4	33.1
**AGO-2 and AGO-4**	25.5	22.7	25.2	8.7
**AGO-5**	32.0	30.3	34.3	50.4

## Discussion

Mixed infections between *Alphanecroviruses* are common in nature [9] and can increase disease severity, transmission, infection, and crop loss. Our study demonstrates that A. *oleae*, a highly virulent virus, facilitates the systemic infection of *A. tessellati*, a less virulent species, by overcoming post-transcriptional gene silencing (PTGS) in *N. benthamiana* [49]. To our knowledge, this is the first report revealing that two closely related viruses, sharing over 74% genome identity, elicit distinct silencing responses during single and mixed infections, including shifts in Dicer-dependent vsiRNAs processing and viral targeting. This finding indicates that genomic similarity alone does not predict host silencing outcomes in mixed-infection contexts.

Single infections showed contrasting phenotypes. *A. oleae* caused rapid and severe systemic symptoms in *N. benthamiana*, affecting all leaves and plants by 10 dpi and leading to high mortality, whereas *A. tessellati* induced only mild, transient symptoms, with plants fully recovering by 8 dpi. In mixed infection, symptom development was accelerated, and disease severity increased compared to single infections. Interestingly, viral accumulation showed divergent trends: *A. tessellati* levels increased significantly under mixed infection, while *A. oleae* abundance declined relative to single infections. These results suggest that *A. oleae* acts as a silencing suppressor, functionally complementing *A. tessellati* and enabling its systemic spread, thereby creating an asymmetric interaction [50–52]. Our findings also reinforce that symptom severity is not always correlated with the abundance of both viruses. This suggests that the two viruses may converge upon host responses or amplify disease via shared signaling pathways, including hormonal or oxidative stress responses. This pattern contrasts with unrelated viral pairs, which often show simultaneous synergistic accumulation, and aligns with observations that related viruses may compete or interact asymmetrically [15, 53–56]. *DCL* analyses are consistent with a higher *DCL4* relative expression in antiviral responses, while *DCL2* may function as a compensatory component [30, 31, 38, 57]. However, in mixed infections, both *DCL4* and *DCL2* were strongly upregulated, suggesting cooperative activation rather than a strict hierarchy. *DCL2* expression exceeded levels observed in single infections, challenging the view of *DCL2* redundancy in the presence of *DCL4*. Qin et al. [58] proposed that *DCL4* and *DCL2* may operate as a dual protective frontline during intra- and intercellular silencing in *N. benthamiana*. Consistently, Deleris et al. [28] demonstrated that increased viral dosage can overcome *DCL2*-mediated effects, although *DCL4* activity does not appear to increase proportionally with viral load. Together, these results suggest regulatory flexibility in *N. benthamiana*, where *DCL2* may act not only as a compensatory factor but also as an active co-participant in antiviral defense when multiple viruses challenge the silencing machinery, particularly in responses to *Alphanecroviruses*.

*DCL3* was transiently induced early in infection, particularly in *A. tessellati* infections, and partially compensated in *dcl2*- or *dcl4*-suppressed plants, reflecting the flexible interplay among *DCLs* to reinforce antiviral silencing under increased viral challenge.

Deep sequencing analyses revealed further complexities in vsiRNA biogenesis. *A. tessellati* generated the highest vsiRNAs levels in single infection, while in the mixed-infection vsiRNA levels decreased by ∼50%. *A. oleae* vsiRNAs dropped nearly 17-fold in mixed infection despite still being present at moderate abundance, indicating that vsiRNA accumulation is not strictly proportional to viral load. This decoupling suggests that virus–virus interactions and modulation of host Dicer processing influence vsiRNA biogenesis.

Both viruses exhibited a strong positive-sense strand bias in vsiRNAs, consistent with previous reports for + ssRNA viruses [59–63], though *A. oleae* showed a slight increase in negative-strand vsiRNAs during mixed infection, possibly reflecting altered replication dynamics or silencing competition.

VsiRNA size profiling revealed a novel shift in *A. oleae* from 20-nt dominance in single infection to 21-nt in mixed infection, a phenomenon not previously reported. This observation may present a novel concept that vsiRNAs length profiles are dynamic and context-dependent, potentially shaped by competitive interactions among DCLs, viral suppressors, or structural features of the viral RNA altered in mixed infection. Since 21-nt vsiRNAs are typically associated with *DCL4* from the same *Tombusviridae* family [43], at first sight this change might indicate a higher efficiency in silencing, however, the response to this better efficiency would be the accumulation of vsiRNAs, but again, the results of *A. oleae* vsiRNAs accumulation in the mixed infection showed the opposite. This shift from 20-nt to 21-nt could also suggest a reconfiguration of *DCL* activity, possibly a reallocation from *DCLs* or, based on the results, a shift in substrate preference due to viral suppressor interference seems the most plausible. Such plasticity in the silencing machinery adds a new layer of complexity to RNAi-based defenses in mixed infections.

20-nt vsiRNAs, consistently abundant across conditions and reported for other viruses [64, 65] may represent non-canonical cleavage products or *DCL* isoform activity, emphasizing gaps in our understanding of *DCL* processing specificity. Hotspot analyses further support this notion of dynamic antiviral response. In both single and mixed *A. tessellati* infections, vsiRNAs predominantly targeted the *RdRp* region establishing a stable hotspot. In contrast, *A. oleae* vsiRNA targeting shifted from the *RdRp* region in single infection to the suppressor-associated p6 gene in mixed infection. This suggests a host-directed redirection of silencing in response to co-infection or potential viral modulation to protect essential replication regions. *A. oleae p6* shares sequence identity with *A. tessellati* *p6*, previously identified as a silencing suppressor, reinforcing the idea that virus–virus interactions can reshape host antiviral targeting.

5’ terminal nucleotide analysis indicated preferential *AGO* loading, with *A. tesselati* vsiRNAs favoring C and U, and *A. oleae* vsiRNAs showing increased C under mixed infection, suggesting selective *AGO* recruitment influenced by viral context. Other studies on RNA viruses observed a higher vsiRNAs with A and U in the 5`-terminal end [38, 39, 66]. Overall, these data reveal that mixed infections alter not only vsiRNA abundance and size but also genomic targeting and potential *AGO* engagement, reflecting a highly plastic and virus-specific silencing response. Our results have practical implications for RNAi-based crop protection. Stable vsiRNA hotspots, such as the *RdRp* region in *A. tessellati*, may serve as targets for transgenic hairpin constructs, topical dsRNA sprays, or CRISPR/Cas13d applications [67]. Understanding how mixed infections reshape silencing efficiency and vsiRNA distribution is essential for predicting antiviral outcomes and designing effective control strategies. In conclusion, this study presents novel insights into how *N. benthamiana*, reconfigures its RNA silencing machinery in response to single and mixed infections by closely related *Alphanecroviruses*. Mixed infection enhances *DCL4* and *DCL2* expression, modifies vsiRNA biogenesis, and redirects silencing targets, creating asymmetric and dynamic virus–host interactions. Taken together, our findings refine understanding of viral synergism and antagonism, highlight the plasticity of plant RNA silencing, and inform strategies for managing mixed viral infections in crops. To further build upon these insights, future high-throughput sequencing studies encompassing expanded biological datasets will be valuable to reinforce the statistical resolution and generalize the host response patterns observed here. Importantly, these findings highlight that subtle shifts in vsiRNA size distribution, target site selection, and *DCL* activity may substantially influence antiviral efficiency, a concept that may extend to other agriculturally important hosts potentially affected by *A. tessellati* and *A. oleae*.

## Material and methods

### Plant growth, virus infections and sampling protocol

Wild-type *N. benthamiana* plants and *N. benthamiana* transgenic lines suppressed for *dcl2* and *dcl4* genes [68, 69] were grown in controlled growth chambers at 25 °C under a 16 h light/8 h dark photoperiod. *DCL*-suppressed lines were used exclusively for virus accumulation and *DCL* gene expression analyses. Viral inocula consisted of purified preparations of *A. tessellati* (GenBank: HQ651834.1) and *A. oleae* isolate GM6 (GenBank: DQ083996.1). For single infections, purified virus preparations were diluted 1:1 (v/v) in 0.05 M sodium phosphate buffer prior to mechanical inoculation. For mixed infections, equal volumes of each purified virus preparation were combined 1:1 (v/v) immediately before inoculation, without further dilution. This approach ensured that each virus was delivered at the same final concentration and individual dose as in the respective single infections, resulting in a higher cumulative viral load in the mixed inoculum. Virus purification was performed as described previously [18]. Briefly, infected leaves were homogenized in cold 0.1 M sodium phosphate buffer (1:3, w/v), filtered, clarified in the presence of organic solvents, concentrated by differential centrifugation, and further purified by ultracentrifugation through sucrose density gradient columns. Virus concentration was determined by measuring absorbance at 260 nm using a Quawell Q9000 micro spectrophotometer (Quawell Technology, Beijing, China) applying an extinction coefficient *E*^1%^_260_ = 5.0. Each plant (one leaf per plant) was mechanically inoculated with 6 µg of purified virus. Four experimental groups were established with 12 biological replicates each (total n = 48): (1) mock-inoculated (buffer only), (2) *A. tessellati*-infected, (3) *A. oleae*-infected, and (4) *A. tessellati* + *A. oleae* mixed-infected. Samples were collected at one day post-inoculation (1dpi; inoculated leaves) and at 5 and 12 dpi from newly emerged, systemically infected leaves located above the inoculated leaves.

At each time point, three independent plants per treatment were sampled, and plants were not resampled at subsequent time points. For each biological replicate, 100 mg of leaf tissue was ground in liquid nitrogen and used for total RNA extraction. Symptom development was monitored daily for up to 30 days post-inoculation.

### Evaluation of disease symptoms

To quantify disease progression, a four-level symptom severity scale was used: 0 = asymptomatic; 1 = mild chlorotic mosaic; 2 = pronounced chlorotic mosaic; 3 = necrotic mosaic; and 4 = extensive leaf necrosis with deformation. Symptom evaluation was performed exclusively on wild-type *N. benthamiana* plants. Infected plants, either singly infected with *A. tessellati* or *A. oleae*, or mixed-infected (both), were observed daily for 30 days. As described by Varanda et al. [2] the disease severity was quantified using the Disease Severity Index (DSI), calculated as: DSI (%) = [Σ (individual disease scores)/(total observations × maximum score)] × 100.

### RNA extraction, RT-qPCR and vsiRNA deep sequencing

Total RNA was extracted from leaf samples (three biological replicates per condition) at 1 dpi, 5 dpi, and 12 dpi using RNeasy Mini kit (Qiagen) according to the manufacturer’s instructions. Purified RNA was resuspended in RNase-free water and immediately stored at − 80 °C until further use. RNA integrity and concentration were assessed using a Quawell 9000 microvolume spectrophotometer.

Specific primers for real-time PCR were designed targeting the *coat protein* (*CP*) regions of *A. tessellati* and *A. oleae*, selected based on their sequence divergence as described by Varanda et al. [9], to enable accurate differentiation between the two viruses. Primer design was performed using Primer Express 3.0 (Applied Biosystems, Foster City, CA, USA), with default parameters, except for the *A. tessellati*d_3 primer, which was adopted from the same previous study. As endogenous reference genes, phosphatase 2 A (PP2) and F-box protein (F-box) were employed (Table 6). Additionally, to investigate the potential involvement of the RNA silencing pathway in viral replication and infectivity, expression levels of *DCL1* to *DCL4* genes were quantified by RT-qPCR in wild-type *N. benthamiana* plants and in transgenic lines suppressed for each *DCL* gene. All primer sequences used in RT-qPCR are listed in Table 1. Reverse transcription was carried out using 1 µg of total RNA in a final volume of 20 µL, employing the Maxima First Strand cDNA Synthesis Kit for RT-qPCR (Thermo Scientific), following the protocol recommended by the manufacturer.

**Table 6:** Primers used in this study for RT–qPCR analysis targeting the coat protein (CP) gene of *A. tessellati* and *A. oleae* in *N. benthamiana* infections

Primer name	Sequence (5’ – 3’)	Position in genome	Region	Reference
CP_*A. tessellati*_5’	CCAAAACATGCCTAAGAGAG	2629—2648	CP	This study
CP *A. tessellati*d_3’	CCTAGATCTTCTGGGCTAAGC	2791—2771	CP	Varanda et al. 2010 [9]
CP_*A. oleae*_5’	TATGAAAGCGGATTTCACCC	2708—2728	CP	This study
CP_*A. oleae*_3’	CTGCTCATTAGTAGTAGTAAT	2877—2857	CP	This study
PP2ArtFW	GACCCTGATGTTGATGTTCGCT	-	-	Liu et al. 2012
PP2ArtREV	GAGGGATTTGAAGAGAGATTTC	-	-	Liu et al. 2012
FBOXrtFW	GGCACTCACAAACGTCTATTTC	-	-	Liu et al. 2012
FBOXrtREV	ACCTGGGAGGCATCCTGCTTAT	-	-	Liu et al. 2012
qNbDCL1-F	AAAAGAATGAGATGGTATTTCGG	-	-	Dadami et al., 2013 [68]
qNbDCL1-R	TTCTTTCTGGCATGCTCAA	-	-	Dadami et al. 2013 [68]
qNbDCL2-F	GAAGAACCACTTCTTAGGGGAAA	-	-	Dadami et al. 2013 [68]
qNbDCL2-R	GGCCATAACAAGGACTCAA	-	-	Dadami et al. 2013 [68]
qNbDCL3-F	AGGACTGCAGCGTTTATGGT	-	-	Katsarou et al. 2016
qNbDCL3-R	GGCCACAACAACCATGTCCT	-	-	Katsarou et al. 2016
DCL4_For b	GCACTTAACTACAGAGAAATGCAATG	-	-	Kotakis et al. 2020
DCL4_Rev b	ACAATGTTTGAGCGCCTTCT	-	-	Kotakis et al. 2010

Quantitative real-time PCR was performed using the Line Gene 9600 Fluorescent Detection System (Bioer). Each reaction contained 12.5 µL of 2 × SYBR Green PCR Master Mix, 0.3 µM of forward and reverse primers, and 12.5 ng of cDNA in a total volume prepared in 96-well plates. Thermal cycling conditions included an initial denaturation at 95 °C for 10 min, followed by 40 cycles of 95 °C for 15 s and 60 °C for 1 min. Negative controls included no-template reactions and cDNA from uninfected plants. All samples were analyzed in triplicate as technical replicates. Ct values were calculated using the fit-point method with fluorescence thresholds manually adjusted above background using the Line Gene 9600 software. Melt curve analysis was conducted at the end of each run to verify primer specificity. Relative expression levels were estimated using standard curves automatically generated by the system, and results were normalized against *PP2* and *F-box* reference genes.

For vsiRNA profiling, only wild-type *N. benthamiana* plants were used. Total RNA from three biological replicates per infection type was pooled prior to small RNA sequencing, precluding independent statistical analysis. Therefore, the vsiRNA profiling results are descriptive. Small RNA sequencing was performed at 5 dpi, a time point that showed optimal viral load and DCL expression, without severe leaf necrosis or deformation. Total small RNA sequencing quality and concentration were assessed by determining the RNA Integrity Number (RIN). Small RNA libraries were constructed using the NEBNext Multiplex Small RNA Library Prep Kit (New England Biolabs), following the manufacturer’s instructions. Sequencing was performed on the Illumina NovaSeq platform, generating 18–36 bp single-end reads. Raw sequencing data corresponding to viral small interfering RNAs (vsiRNAs) were processed and quality-trimmed using CLC Genomics Workbench v12.0.3 (Qiagen, 2019). High-quality reads were mapped against the complete genomes of *A. tessellati* and *A. oleae* (GenBank accessions HQ651834.1 and DQ083996.1, respectively), using stringent parameters (length and similarity fractions set to 1.0) to ensure 100% sequence alignment. Only small RNAs with read counts ≥ 100 were retained for downstream analyses. Annotation was performed using a curated, non-redundant plant virus database (vrl_plant v232; [70]) and the miRBase v22.1 database [71] to classify known and novel small RNAs.

### Statistical data analysis

Pairwise sequence identities of the full genomes of the two *Alphanecroviruses* representatives used in this work were determined using the Job Dispatcher bioinformatics tool at EMBL-EBI [72]. For nucleotide sequence alignment of both viruses, the Clustal Omega alignment program (European Bioinformatics Institute, EMBL-EBI) was used. PRIMER v6 software [73] and PERMANOVA add-on package [74] were used to perform univariate and multivariate analyses with the permutational analysis of variance to detect significant differences (p < 0.05): in the viruses' relative abundance and *DCLs* relative expression levels between: “Treatment” single *A. tessellati*-infection, single *A. oleae*-infection and *A. tessellati* and *A. oleae* mixed-infection and “Time points” 1 dpi, 5 dpi, and 12 dpi. To test the null hypotheses that no significant differences existed, a two-way PERMANOVA was applied between; “Treatment” and “Time points”. PERMANOVA analyses were carried out with the following two-factor design: “Treatment” single *A. tessellati*-infection, single *A. oleae*-infection and *A. tessellati* and *A. oleae* mixed-infection (three levels, fixed); and “Time points”, 1 dpi, 5 dpi, and 12 dpi (three levels, random). In addition, in the *DCLs* analysis it was included uninfected (only buffer inoculated) plants at the “Treatment” level. The data were square root transformed in order to scale down the importance of the high values of relative viruses and *DCLs* levels. PERMANOVA was applied using a Bray–Curtis similarity matrix, following the method of Clarke and Green [74]. When the number of unique permutations was lower than 150, p-values were adjusted using the Monte Carlo test. In cases where significant interaction effects were identified, pairwise comparisons were subsequently performed with 9999 permutations under a reduced model to assess differences between groups [75].

## Supplementary Information

Below is the link to the electronic supplementary material.

Supplementary Material 1. Table S1. The two-way PERMANOVA test conducted between; “Treatment” (mock-inoculated, single *A. tessellati*-infection, single *A. oleae*-infection *A. tessellati* and *A. oleae* mixed-infection) and “Time points” (1dpi, 5dpi and 12 dpi) using pairwise comparisons performed with 9999 permutations under a reduced model to assess differences between DCLs (DCL1–DCL4) genes in *dcl2 *and *dcl4*-suppressed in* N. benthamiana* plants.

Supplementary Material 2. Table S2. Summary of the total number of reads and their respective percentages in the non-coding regions at each type of infection (single *A. tessellati*-infection, single *A. oleae*-infection, *A. tessellati* and *A. oleae* mixed-infection).

## Data Availability

The vsiRNA datasets generated and analyzed during the current study are available in the NCBI Sequence Read Archive (SRA) repository under BioProject accession PRJNA1401704 (https://www.ncbi.nlm.nih.gov/bioproject/PRJNA1401704). The datasets correspond to vsiRNAs obtained from Nicotiana benthamiana plants subjected to single infection with A. tessellati (OMMV) (BioSample SAMN54572195: https://www.ncbi.nlm.nih.gov/sra/SRX31778995 [accn]), single infection with A. oleae (OLV-1) (BioSample SAMN54572196: https://www.ncbi.nlm.nih.gov/sra/SRX31778996 [accn]), mixed infection with A. tessellati and A. oleae (OMMV and OLV-1) (BioSample SAMN54572197: https://www.ncbi.nlm.nih.gov/sra/SRX31778997 [accn]), and virus-free control plants (BioSample SAMN54572198: https://www.ncbi.nlm.nih.gov/sra/SRX31778998 [accn]).
