# Supplementary material for: Mixed viral infections uncover multifaceted RNA silencing strategies in plant antiviral defense
Source: BMC Genomics. 2026 Jul 7;27:804. doi: 10.1186/s12864-026-13141-8 (PMC13628807; doi:10.1186/s12864-026-13141-8)
Supplement: Supplementary file 1 [file 12864_2026_13141_MOESM1_ESM.docx]

| **Treatments** | **DCL1** | | | **DCL3** | | | **DCL4** | | | | |
| --- | --- | --- | --- | --- | --- | --- | --- | --- | --- | --- | --- |
| **DCL2 supressed** | 1dpi | 5dpi | 12 dpi | 1dpi | 5dpi | 12 dpi | 1dpi | 5dpi | 12 dpi |  |  |
|  | *p* values | | | *p* values | | | *p* values | | | | |
| mock-inoculated *vs* single-*A. tessellati* | 0,0239 | 0,0001 | 0,001 | 0,003 | 0,0003 | 0,0229 | 0,0001 | 0,0001 | 0,0001 |  |  |
| mock-inoculated *vs* single-*A. oleae* | 0,0438 | 0,0008 | 0,6941 | 0,0006 | 0,0055 | 0,0001 | 0,0016 | 0,01 | 0,5041 |  |  |
| mock-inoculated *vs* mixed infection | 0,0241 | 0,0011 | 0,1083 | 0,0003 | 0,4082 | 0,7423 | 0,0001 | 0,0001 | 0,0001 |  |  |
| single-*A. tessellati* *vs* single-*A. oleae* | 0,5135 | 0,0002 | 0,0004 | 0,0818 | 0,0001 | 0,0007 | 0,0001 | 0,0001 | 0,0002 |  |  |
| single-*A. tessellati* *vs* mixed infection | 0,6319 | 0,0036 | 0,0035 | 0,0006 | 0,0001 | 0,0295 | 0,0002 | 0,0001 | 0,0001 |  |  |
| single-*A. oleae* *vs* mixed infection | 0,3887 | 0,0746 | 0,106 | 0,0001 | 0,0003 | 0,0001 | 0,0001 | 0,0001 | 0,0001 |  |  |
|  |  |  |  |  |  |  |  |  |  |  |  |
| **Treatments** | **DCL1** | | | **DCL3** | | | **DCL4** | | | | |
| **DCL2 supressed** | 1 dpi *vs* 5 dpi | 1 dpi *vs* 12 dpi | 5 dpi *vs* 12 dpi | 1 dpi *vs* 5 dpi | 1 dpi *vs* 12 dpi | 5 dpi *vs* 12 dpi | 1 dpi *vs* 5 dpi | 1 dpi *vs* 12 dpi | 5 dpi *vs* 12 dpi |  |  |
|  | *p* values |  |  | *p* values |  |  | *p* values | | |  |  |
| mock-inoculated | 0,1834 | 0,8006 | 0,2556 | 0,6484 | 0,1612 | 0,7829 | 0,0327 | 0,0006 | 0,6351 |  |  |
| single-*A. tessellati* | 0,0012 | 0,0018 | 0,0623 | 0,0002 | 0,0737 | 0,0001 | 0,0001 | 0,0001 | 0,0001 |  |  |
| single-*A. oleae* | 0,0666 | 0,0178 | 0,0599 | 0,0223 | 0,0031 | 0,4227 | 0,0183 | 0,4181 | 0,0089 |  |  |
| mixed infection | 0,3821 | 0,1438 | 0,3931 | 0,0001 | 0,0001 | 0,0896 | 0,0001 | 0,0001 | 0,0015 |  |  |
| **Treatments** | **DCL1** | | | **DCL2** | | | **DCL3** | | | | |
| **DCL4 supressed** | 1 dpi | 5 dpi | 12 dpi | 1 dpi | 5 dpi | 12 dpi | 1 dpi | 5 dpi | 12 dpi |  |  |
|  | *p* values | | | | | | | | | |  |
| mock-inoculated *vs* single-*A. tessellati* | 0,0022 | 0,0087 | 0,0001 | 0,0001 | 0,0001 | 0,0001 | 0,0011 | 0,0001 | 0,0001 |  |  |
| mock-inoculated *vs* single-*A. oleae* | 0,0003 | 0,0004 | 0,0002 | 0,0002 | 0,0001 | 0,0001 | 0,595 | 0,1562 | 0,0008 |  |  |
| mock-inoculated *vs* mixed infection | 0,0001 | 0,0002 | 0,0001 | 0,0001 | 0,0001 | 0,0001 | 0,0001 | 0,0001 | 0,0001 |  |  |
| single-*A. tessellati* *vs* single-*A. oleae* | 0,0734 | 0,0009 | 0,0001 | 0,0001 | 0,0681 | 0,1527 | 0,0071 | 0,0001 | 0,0001 |  |  |
| single-*A. tessellati* *vs* mixed infection | 0,0009 | 0,0002 | 0,0002 | 0,0006 | 0,3186 | 0,0024 | 0,0002 | 0,6432 | 0,136 |  |  |
| single-*A. oleae* *vs* mixed infection | 0,0008 | 0,0001 | 0,0001 | 0,0001 | 0,0084 | 0,0119 | 0,0001 | 0,0001 | 0,0001 |  |  |
|  |  |  |  |  |  |  |  |  |  |  |  |
| **Treatments** | **DCL1** | | | **DCL2** | | | **DCL3** | | | | |
| **DCL4 supressed** | 1 dpi *vs* 5 dpi | 1 dpi *vs* 12 dpi | 5 dpi *vs* 12 dpi | 1 dpi *vs* 5 dpi | 1 dpi *vs* 12 dpi | 5 dpi *vs* 12 dpi | 1 dpi *vs* 5 dpi | 1 dpi *vs* 12 dpi | 5 dpi *vs* 12 dpi |  |  |
|  | *p* values | | | | | | | | | |  |
| mock-inoculated | 0,0036 | 0,0066 | 0,2377 | 0,2151 | 0,0201 | 0,0911 | 0,0018 | 0,0001 | 0,0019 |  |  |
| single-*A. tessellati* | 0,8935 | 0,0001 | 0,0002 | 0,0001 | 0,0004 | 0,0001 | 0,0001 | 0,0001 | 0,0801 |  |  |
| single-*A. oleae* | 0,1164 | 0,0636 | 0,3991 | 0,0001 | 0,0001 | 0,0001 | 0,1415 | 0,5772 | 0,1683 |  |  |
| mixed infection | 0,0139 | 0,0017 | 0,0923 | 0,0001 | 0,0013 | 0,0001 | 0,0001 | 0,0001 | 0,0088 |  |  |
