# Supplementary material for: Mixed viral infections uncover multifaceted RNA silencing strategies in plant antiviral defense
Source: BMC Genomics. 2026 Jul 7;27:804. doi: 10.1186/s12864-026-13141-8 (PMC13628807; doi:10.1186/s12864-026-13141-8)
Supplement: Supplementary file 2 [file 12864_2026_13141_MOESM2_ESM.docx]

|  | genome position | 2440–2442 | 2614–2635 | 3446–3683 | total non-coding reads |
| --- | --- | --- | --- | --- | --- |
| *A. tessellati* | Nº of reads | 6171 | 15 841 | 201 072 | 510 865 |
|  | % | **0.1** | **0.3** | **3.4** | **8.8** |
| *A. tessellati*-mixed | Nº of reads | 2215 | 6031 | 76 321 | 213 314 |
|  | % | **0.1** | **0.2** | **3.0** | **8.5** |
|  | genome position | 2441–2442 | 2614–2635 | 3446–3683 |  |
| *A. oleae* | Nº of reads | 825 | 1249 | 43 292 | 100 522 |
|  | % | **0.1** | **0.1** | **3.4** | **7.8** |
| *A. oleae*-mixed | Nº of reads | 397 | 360 | 1325 | 7049 |
|  | % | **0.5** | **0.5** | **1.7** | **9.2** |
